# Insight into the role of α-arabinofuranosidase in biomass hydrolysis: cellulose digestibility and inhibition by xylooligomers

**DOI:** 10.1186/s13068-019-1412-0

**Published:** 2019-03-22

**Authors:** Donglin Xin, Xiang Chen, Peiyao Wen, Junhua Zhang

**Affiliations:** College of Forestry, Northwest A&F University, 3 Taicheng Road, Yangling, 712100 Shaanxi China

**Keywords:** Biomass, Kinetics, α-Arabinofuranosidase, Inhibition, Xylooligomers

## Abstract

**Background:**

α-l-Arabinofuranosidase (ARA), a debranching enzyme that can remove arabinose substituents from arabinoxylan and arabinoxylooligomers (AXOS), promotes the hydrolysis of the arabinoxylan fraction of biomass; however, the impact of ARA on the overall digestibility of cellulose is controversial. In this study, we investigated the effects of the addition of ARA on cellulase hydrolytic action.

**Results:**

We found that approximately 15% of the xylan was converted into AXOS during the hydrolysis of aqueous ammonia-pretreated corn stover and that this AXOS fraction was approximately 12% substituted with arabinose. The addition of ARA removes a portion of the arabinose decoration, but the resulting less-substituted AXOS inhibited cellulase action much more effectively; showing an increase of 45.7%. Kinetic experiments revealed that AXOS with a lower degree of arabinose substitution showed stronger affinity for the active site of cellobiohydrolase, which could be the mechanism of increased inhibition.

**Conclusions:**

Our findings strongly suggest that the ratio of ARA and other xylanases should be carefully selected to avoid the strong inhibition caused by the less-substituted AXOS during the hydrolysis of arabinoxylan-containing biomass. This study advances our understanding of the inhibitory mechanism of xylooligomers and provides critical new insights into the relationship of ARA addition and cellulose digestibility.

**Electronic supplementary material:**

The online version of this article (10.1186/s13068-019-1412-0) contains supplementary material, which is available to authorized users.

## Background

Biofuels from lignocellulosic biomass have been considered a promising alternative to traditional fossil fuels [1–3]. However, the conversion process is not cost-effective because the cost of the conversion process is high and the biofuels productivity is low, which makes the price of biofuels too high to compete with traditional fossil fuels [4]. To address this issue, numerous research studies have focused on how to economically produce biofuels from lignocellulosic biomass [5–7]. One way of improving the efficiency of biofuels production is to digest xylan, which can significantly improve access of cellulose to cellulases and enhance the hydrolysis yield of cellulose in xylan-containing lignocellulosic biomass [5]. In addition, xylose could be fermented to ethanol in parallel with glucose, which provides additional benefits to the biofuels production process [8]. Therefore, xylan digestion and its resulting effects on cellulose hydrolysis, cellulase action, and fermentable sugar yields have attracted considerable research interest during the previous decade [9, 10].

As previously reported, there was a nearly linear relationship between the increases in xylose and glucose yields during the hydrolysis of biomass after pretreatment by many technologies, such as ammonia fiber expansion, ammonia recycle, and hydrothermal [11, 12]. This finding clearly shows the significance of completely converting xylan into xylose for the highly effective hydrolysis of lignocellulosic biomass. However, it is difficult to achieve this goal because the structure of xylan is extremely complex. In graminaceous monocots, for example, arabinoxylan is the major hemicellulosic polymer. Arabinoxylan is most commonly substituted by a single α-l-arabinofuranosyl unit on either the C2- or C3-position of xylose, or both the C2- and C3-positions of xylose [13, 14]. Furthermore, its β-D-xylopyranose main chain residues are also substituted with many other chemistries, including acetyl groups and 4-*O*-methyl-glucuronopyranosyl groups [15, 16]. Theoretically, the complete hydrolysis of xylan requires the synergistic action of several enzymes: endoxylanases which depolymerize the xylan backbone to xylooligomers and debranching enzymes; such as α-glucuronidase, α-l-arabinofuranosidase (ARA), and acetyl esterase to remove the corresponding side groups; and β-xylosidases which hydrolyze xylooligomers to xylose from its non-reducing end. Single arabinofuranosyl substitutions that decorate β-D-xylopyranose residues can be removed by ARA of Glycoside Hydrolase Family 51, 54 or 62 (GH51, GH54 or GH62); arabinofuranose of di-substituted β-D-xylopyranose residues can be removed by GH 43 ARA [17, 18]. However, the contents of these arabinose-debranching enzymes are low in many common commercial cellulase and xylanase preparations, such as Cellic CTEC, Htec, Accellerase 1500, and Celluclast 1.5L [19–21]. Therefore, the effect of adding extra ARA on the conversion of lignocellulosic biomass into monosaccharides has attracted intensive research interest [11, 20, 22].

The addition of ARA has shown increase in xylose yields, but whether ARA can significantly increase the hydrolysis of cellulose in lignocellulosic biomass remains controversial. Alvira et al. reported that the addition of ARA to the mixture of cellulase and endoxylanase resulted in increased conversion of cellulose in steam explosion-pretreated wheat straw into glucose [20]. However, Gao et al. and Zhang et al. reported no effect from the addition of ARA on the hydrolysis of cellulose in ammonia fiber expansion-pretreated corn stover and hydrothermally pretreated wheat straw [11, 22]. In addition, Seling et al. found that after debranching by ARA, wheat arabinoxylan inhibited cellobiohydrolase more strongly with a notable increase of 50% in the inhibitory capacity [23], indicating that ARA may negatively affect cellulase action. These results prompted us to initiate an additional study on the role of ARA in the hydrolysis of arabinoxylan-containing biomass and to determine whether ARA addition will intensify the inhibition of xylooligomers on cellulase.

Compared to xylan, xylooligomers show a much stronger inhibitory effect on cellulase activity and present a major cause of the loss in cellulase activity [24]. It was also found that approximately 20% of the xylan was converted into xylooligomers during the hydrolysis of AFEX-pretreated corn stover (15% solid loading) with the loading of 15 mg/g enzyme (Ctec2:Htec2:Multifect Pectinase, 1:1:1) [25]. Such xylooligomers are also found to be subject to substitution by arabinose, uronic acids, and/or acetic acid [26]. Our research and that of others has shown that xylooligomers competitively inhibit cellobiohydrolase I. Furthermore, xylooligomers with long chains are stronger inhibitors than those with shorter chains [27–29]. However, in these studies, the substitution of xylooligomers was not examined. To the best of our knowledge, whether substitution of xylooligomers can affect the inhibition of cellulases is still unknown. Identifying the effect of ARA on xylooligomer inhibition will help us to fully evaluate the role of ARA in the hydrolysis of arabinoxylan-containing materials and thus design cost-effective enzyme mixtures to degrade biomass.

In this study, we investigated the role of additional ARA on the conversion of cellulose and xylan in ammonia aqueous-pretreated corn stover into fermentable monosaccharides. The formation and composition of xylooligomers during the hydrolysis process were tested. The inhibitory effects of xylooligomers on the commercial cellulase, CTec2, and the thermostable individual cellulases, including endoglucanase, cellobiohydrolase, and β-glucosidase, were compared before and after debranching by ARA. The kinetics of the inhibition of cellobiohydrolase by xylooligomers with or without substitution was analyzed to elucidate the mechanism behind the different inhibitory abilities.

## Results

### Debranched AXOS inhibited cellulase more strongly

To assess whether substitution affects the inhibition of AXOS on cellulase, AXOS with or without debranching enzyme was added to the hydrolysis of Avicel. Two different types of cellulase, namely, commercial cellulase (CTec2) and a mixture of thermostable cellulases (CEL), were used in this study to compare their effectiveness. The amount of monosaccharides in the AXOS preparation was measured by HPLC. No xylose or arabinose was observed, indicating that all of the arabinose substituents were linked to the AXOS. To determine the content of the arabinose substituent, the AXOS fraction was treated with 4% sulfuric acid as described by the NREL [30]. The results showed that the ratio of arabinose to xylose was 41:59, indicating that the AXOS was highly substituted with arabinose. Debranched AXOS was obtained by adding ARA to the AXOS preparation. HPLC data revealed that ARA could remove approximately 45% of the arabinose substituent.

**Fig. 1:**
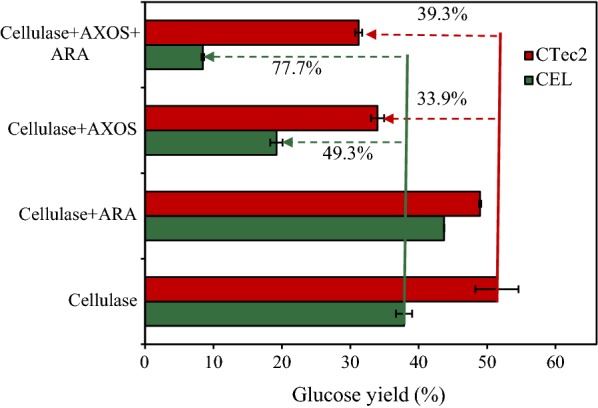
Effect of less-substituted AXOS on hydrolytic action of cellulases. Hydrolysis of 2% Avicel by CTec2 (10 FPU/g DM) and CEL (2 mg/g DM Cel5A, 8 mg/g DM Cel7A, and 0.2 mg/g DM Cel3A) with the addition of AXOS (5 mg/mL) and/or ARA (2 mg/g DM) at 50 °C for 48 h. The error bars represent the standard error of three independent experiments

The results in Fig. 1 show that the addition of ARA clearly intensified the inhibition of AXOS on the hydrolysis of biomass. The degree of inhibition of the AXOS on Avicel hydrolysis increased from 33.9% using CTec2 and 49.3% using CEL to 39.3% and 77.7%, respectively. To determine the fate of the AXOS during the hydrolysis process, the amounts of xylose and arabinose released were determined (Table 1). When adding AXOS to the hydrolysis system, some arabinose (0.2 mg/mL by CTec2 or CEL) was released, indicating that some ARA activity is present in the CTec2 and CEL preparations.

**Table 1:** The formation of xylose and arabinose by XYL and ARA

Enzymes	CTec2		CEL	
	Xylose (mg/mL)	Arabinose (mg/mL)	Xylose (mg/mL)	Arabinose (mg/mL)
Cellulase	0.30 ± 0.1	0.01 ± 0.0	0.01 ± 0.0	0.01 ± 0.0
Cellulase + AXOS	1.13 ± 0.2	0.20 ± 0.0	0.34 ± 0.1	0.20 ± 0.0
Cellulase + AXOS + ARA	2.01 ± 0.1	1.02 ± 0.2	0.41 ± 0.2	0.33 ± 0.2
Cellulase + ARA	0.20 ± 0.0	0.01 ± 0.0	0.02 ± 0.0	0.01 ± 0.0

Hydrolysis of Avicel (10 mg/mL) by different cellulolytic and xylanolytic enzyme preparations (Fig. 1) with the addition of AXOS (5 mg/mL) at 50 °C for 48 h. The error bars represent the standard errors of three experiments

**Fig. 2:**
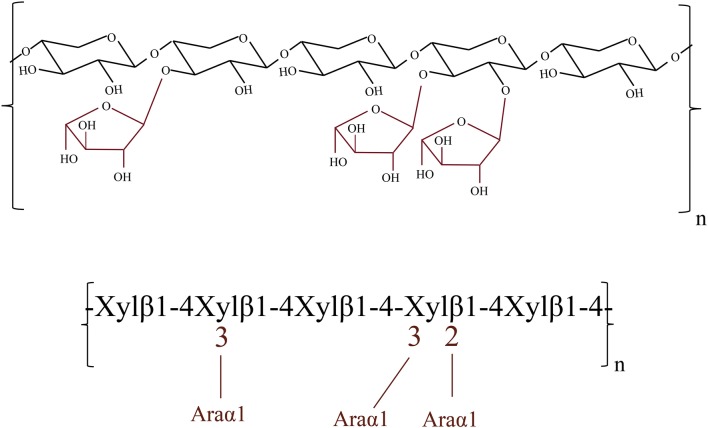
Schematic representation of the structure of arabinoxylan

In addition, some xylose (1.13 mg/mL by CTec2 and 0.34 mg/mL by CEL) was detected, showing that a considerable amount of endoxylanase and/or β-xylosidase activity is present in CTec2, while only small amounts of endoxylanase and/or β-xylosidase activity are present in the thermostable CEL mixture, which agrees well with previous results on the composition of these cellulase preparations [31, 32]. After further supplementation of ARA, sizeable amounts of arabinose (approximately 40–50% of the theoretical amount) were released, and the amounts of xylose released increased, showing that a considerable amount of AXOS was debranched and/or degraded. In addition, the ARA used in this work was found to preferentially hydrolyze arabinofuranose that is singly substituted to β-D-xylopyranose residues. ARA also acts slowly on the arabinofuranose residues of the di-substituted β-D-xylopyranose sugars [33]. Based on the structural information of arabinoxylan (Fig. 2), it could be speculated that ARA mainly removed α-1,3-l-arabinofuranose from β-D-xylopyranose residues and that most of the residual arabinofuranoses released were doubly substituted to β-D-xylopyranose backbone sugars.

In this work, the addition of ARA to AXOS resulted in the release of xylose (less than or equal to 2 mg/mL), arabinose (less than or equal to 1 mg/mL), and less-substituted AXOS in the hydrolysate. Our previous results have confirmed that such a low amount of xylose showed no inhibitory effect on Cel5A, Cel7A, and Cel3A [28]. The results in Additional file 1: Figure S1 further confirmed that arabinose (0.2–5 mg/mL) had no inhibitory effect on hydrolysis of Avicel by CTec2 or CEL. We, thus, hypothesized that debranching significantly increased the inhibitory effect of the AXOS, thus offsetting the positive effect of AXOS degradation, which could be the possible reason for this observation.

### The role of ARA in the hydrolysis of AA-CS

**Fig. 3:**
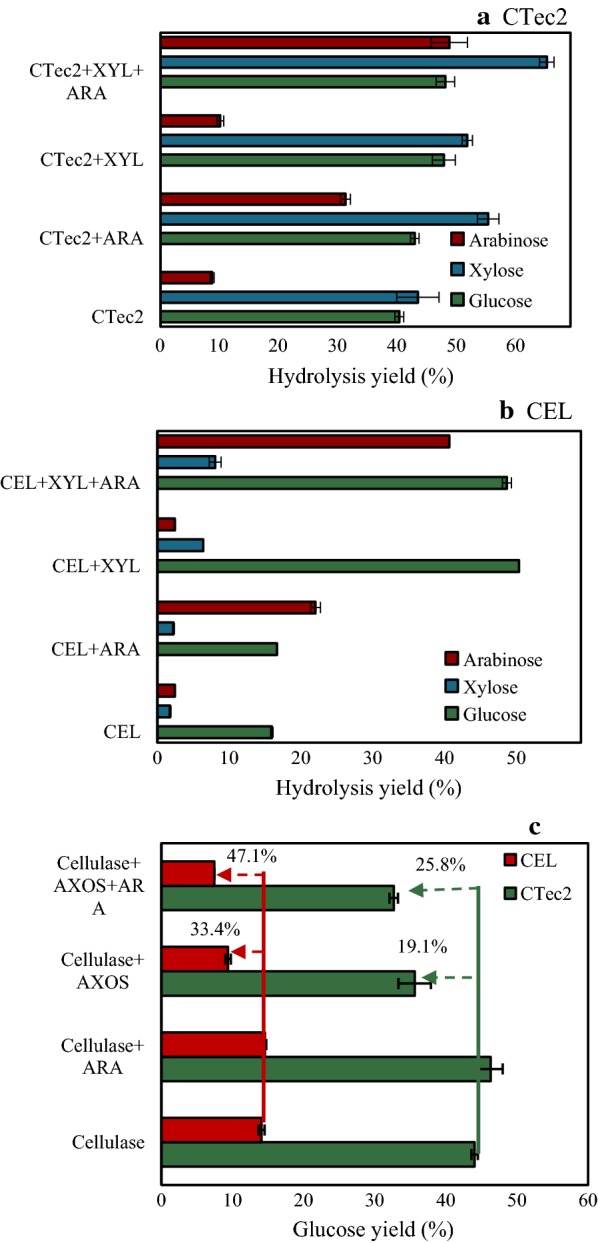
Role of ARA in the hydrolysis of pretreated corn stover. Hydrolysis of 10% corn stover after pretreatment with aqueous ammonia (AA-CS) by CTec2 (10 FPU/g DM) (**a**), or CEL (2 mg/g DM Cel5A, 8 mg/g DM Cel7A, and 0.2 mg/g DM Cel3A) (**b**), and XYL (2 mg/g DM), and/or ARA (2 mg/g DM) at 50 °C for 48 h. Hydrolysis of 10% AA-CS by CTec2 (10 FPU/g DM) and CEL (2 mg/g DM Cel5A, 8 mg/g DM Cel7A, and 0.2 mg/g DM Cel3A) with the addition of AXOS (4 mg/mL) and/or ARA (2 mg/g DM) at 50 °C for 48 h (**c**). The error bars represent the standard error of three independent experiments

The pretreatment of corn stover with aqueous ammonia, a commonly used pretreatment technology that can retain most of the xylan, was used to investigate the effect of ARA during the hydrolysis of the lignocellulosic biomass (Fig. 3a, b). As expected, the addition of XYL resulted in an unambiguous increase in the hydrolysis yield of cellulose in AA-CS, supporting previous results that the solubilization of xylan can significantly increase the hydrolysis of cellulose in the xylan-containing material [34, 35]. However, further addition of ARA showed no effect on cellulose hydrolysis, although a sizable increase in the xylose yields of AA-CS was observed. This shows that the addition of the debranching enzyme ARA did not correlate with the enhancement of cellulose conversion under these conditions. This finding is somewhat surprising because converting xylan and xylooligomers into the end-product xylose has been reported to be an effective way to improve cellulase hydrolytic action. Moreover, a nearly linear relationship between the increases in the yields of xylose and glucose in the hydrolysis of xylan-containing biomass was observed [5–7]. There could be a number of reasons for this phenomenon, but based on the result in Fig. 1, we hypothesized that one of the possible reasons could be that the addition of ARA removed the arabinose groups and generated more inhibitory AXOS, which offsets the positive effects of removing xylooligomers. We, therefore, measured the composition of the AXOS present in the hydrolysate using the method described in the carbohydrate analysis section. During the hydrolysis of AA-CS by CEL, 0.14 mg/mL xylooligomers was released (likely due to the presence of xylanolytic enzymes in the CEL preparations [26]). After further addition of ARA, the amounts of xylooligomers released increased to 1.26 mg/mL. Addition of ARA would be expected to remove arabinose groups of the arabinoxylan, which will increase the sites for endoxylanase hydrolysis ultimately yielding more xylooligomers. However, during the hydrolysis of AA-CS by CTec2, the amounts of AXOS released decreased after addition of ARA. It was found that CTec2 contains a considerable amount of β-xylosidase [32], which could further convert xylooligomers into monosaccharides and result in a decrease in the amount of xylooligomers released. It was observed that approximately 4.0 and 1.5 mg/mL of xylan oligomers were accumulated when AA-CS was hydrolyzed by CTec2 plus XYL and CEL plus XYL, respectively (Table 2). Approximately, 12.3% and 18.3% of these AXOS were substituted with arabinose. After further addition of ARA, the amounts of AXOS that accumulated clearly decreased. In addition, the arabinose content in these fractions decreased to 4.15% and 10.7%, respectively.

**Table 2:** Concentration of AXOS (expressed as monomeric equivalents) in hydrolysate of aqueous ammonia-pretreated corn stover (AA-CS)

	CTec2		CEL	
	Xylose (mg/mL)	Arabinose (mg/mL)	Xylose (mg/mL)	Arabinose (mg/mL)
Cellulase	2.64 ± 0.3	0.30 ± 0.0	0.14 ± 0.0	0.00 ± 0.0
Cellulase + ARA	1.94 ± 0.1	0.21 ± 0.1	1.26 ± 0.2	0.00 ± 0.0
Cellulase + XYL	3.97 ± 0.5	0.56 ± 0.2	1.56 ± 0.3	0.35 ± 0.1
Cellulase + XYL + ARA	2.77 ± 0.2	0.12 ± 0.0	0.75 ± 0.1	0.09 ± 0.0

Hydrolysis of AA-CS by different cellulolytic and xylanolytic enzyme preparations (Fig. 3a, b) at 50 °C for 48 h

To mimic this possible inhibitory effect, an AXOS preparation (4 mg/mL, equal to that detected in the hydrolysis of AA-CS by CTec2 and XYL) was added to the hydrolytic system of AA-CS (Fig. 3c). As expected, further addition of ARA resulted in the increased inhibitory effect of AXOS on the hydrolysis of AA-CS by CTec2 (i.e., 35.1%) and an increase in that of CEL by 41.0%. Even though the amount of the arabinose group in the added AXOS preparation is inconsistent with that in the xylan oligomers derived from the hydrolysis process, these results still provide support for our hypothesis that ARA addition generated stronger inhibitory xylan and xylan oligomers, and therefore, there is no positive effect on cellulose conversion.

### The mechanism behind the inhibition

**Fig. 4:**
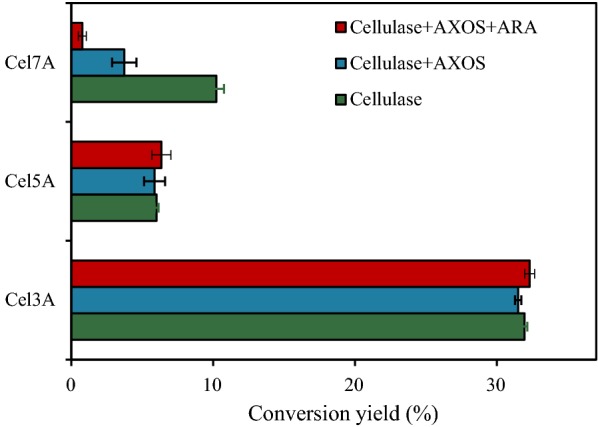
Hydrolytic action of individual cellulase in the presence of AXOS and/or ARA. Hydrolysis of *p*NPC (5 mM) by 8 mg/g DM Cel7A, HEC (10 mg/mL) by 2 mg/g DM Cel5A, and cellobiose (10 mg/mL) by 0.2 mg/g DM Cel3A, with the addition of AXOS (5 mg/mL) and/or ARA (2 mg/g DM) at 50 °C for 1 h. The error bars represent the standard error of three independent experiments

The effects of ARA addition on the inhibition of AXOS on the individual cellulases, Cel5A, Cel7A, and Cel3A were evaluated to thoroughly understand the role of ARA addition on cellulase activity (Fig. 4). The data show that before and after AXOS released some arabinose from the action of ARA, it exhibited no inhibitory effect on Cel5A and Cel3A, but strongly inhibited Cel7A. In addition, the inhibitory ability of AXOS for Cel7A significantly increased by 45.7% from 63.5 to 92.3% when ARA was added, indicating that most of the Cel7A activity was inactivated by this less-substituted AXOS. Selig et al. previously reported that the treatment of wheat arabinoxylan with ARA can increase the inhibitory nature of xylan on Cel7A by approximately 50% [23]. Combined with the results of this study, it could be concluded that the negative effect resulting from the addition of ARA could primarily be attributed to the increased inhibition of Cel7A by xylan and its derivatives which lowered the hydrolytic action of cellulase.

**Fig. 5:**
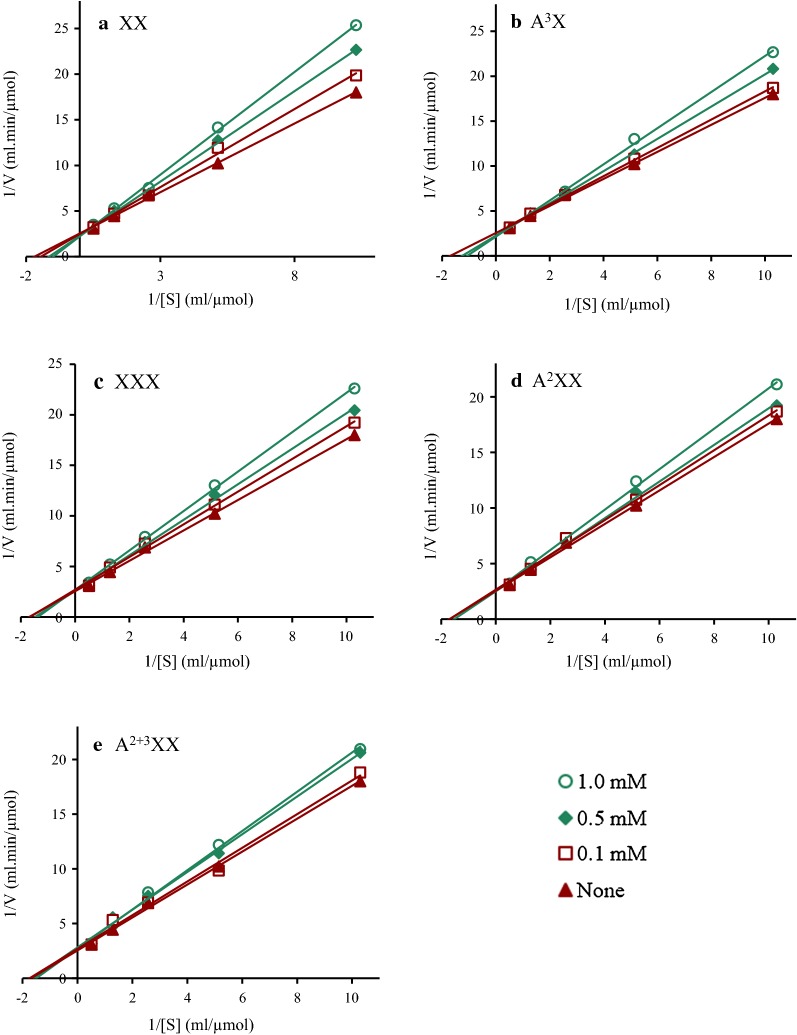
Lineweaver–Burk plots of Cel7A activities at different XX (**a**), A^3^X (**b**), XXX (**c**), A^2^XX (**d**), and A^2+3^XX (**e**) (0.1, 0.5, and 1.0 mM) concentrations. The determination was carried out at 50 °C and pH 5.0 using *p*NPC as substrate

Kinetic experiments were performed to explore the mechanism behind the varying inhibition effect of AXOS on Cel7A with or without an arabinose group in more detail. Instead of the prepared AXOS, pure xylobiose (XX), xylotriose (XXX), and the corresponding xylooligomers with arabinose groups, 3^2^-α-l-Arabinofuranosyl-xylobiose (A^3^X), 2^3^-α-l-arabinofuranosyl-xylotriose (A^2^XX), and 2^3^,3^3^-di-α-l-arabinofuranosyl-xylotriose (A^2+3^XX), were used in these experiments as comparisons (Fig. 5). All the data lines on the Lineweaver–Burk plots intersected on the horizontal axis, and the value of the vertical axis intercept decreased with the increase in the concentrations of these xylooligomers, revealing that xylooligomers with or without arabinose groups are both competitive inhibitors of Cel7A.

The *K*_*m*_ and *K*_*i*_ values were calculated to quantify the degree of inhibition (Table 3). It was observed that xylooligomers with arabinose groups resulted in smaller *K*_*m*_ values by comparing *K*_*m*_ values of XX with A^3^X, and XXX with A^2^XX, whereas more arabinose groups resulted in much smaller *K*_*m*_ values by comparing the *K*_*m*_ values of A^2^XX with A^2+3^XX. The inhibition constant *K*_*i*_ of XXX on Cel7A was 3.22 mM, which was lower than the *K*_*i*_ values of A^2^XX (4.51 mM) (*P *< 0.05) and A^2+3^XX (7.57 mM) (*P *< 0.01). These results further confirmed that xylooligomers without an arabinose group tended to be more easily and firmly bound to the active site of Cel7A, and thus showed a stronger inhibitory effect on Cel7A compared with those that were fully substituted.

**Table 3:** The effects of XX, A^3^X, XXX, A^2^XX, and A^2+3^XX on the $K_{m}^{\text{app}}$ and *K*_*i*_ values of Cel7A using *p*NPC as substrate

Inhibitors	Concentrations (mM)	*K*_*m*_ or $K_{m}^{\text{app}}$ (mM)	*K*_*i*_ (mM)
None	0	0.58	
XX	1.0	1.01	0.98 ± 0.22
	0.5	0.87	
	0.1	0.68	
A^3^X	1.0	0.94	1.62 ± 0.18
	0.5	0.80	
	0.1	0.61	
XXX	1.0	0.73	3.22 ± 0.32
	0.5	0.68	
	0.1	0.60	
A^2^XX	1.0	0.70	4.51 ± 0.85
	0.5	0.68	
	0.1	0.59	
A^2+3^XX	1.0	0.66	7.57 ± 1.12
	0.5	0.61	
	0.1	0.59	

The value of *K*_m_ was obtained with the reaction without inhibitors

## Discussion

**Fig. 6:**
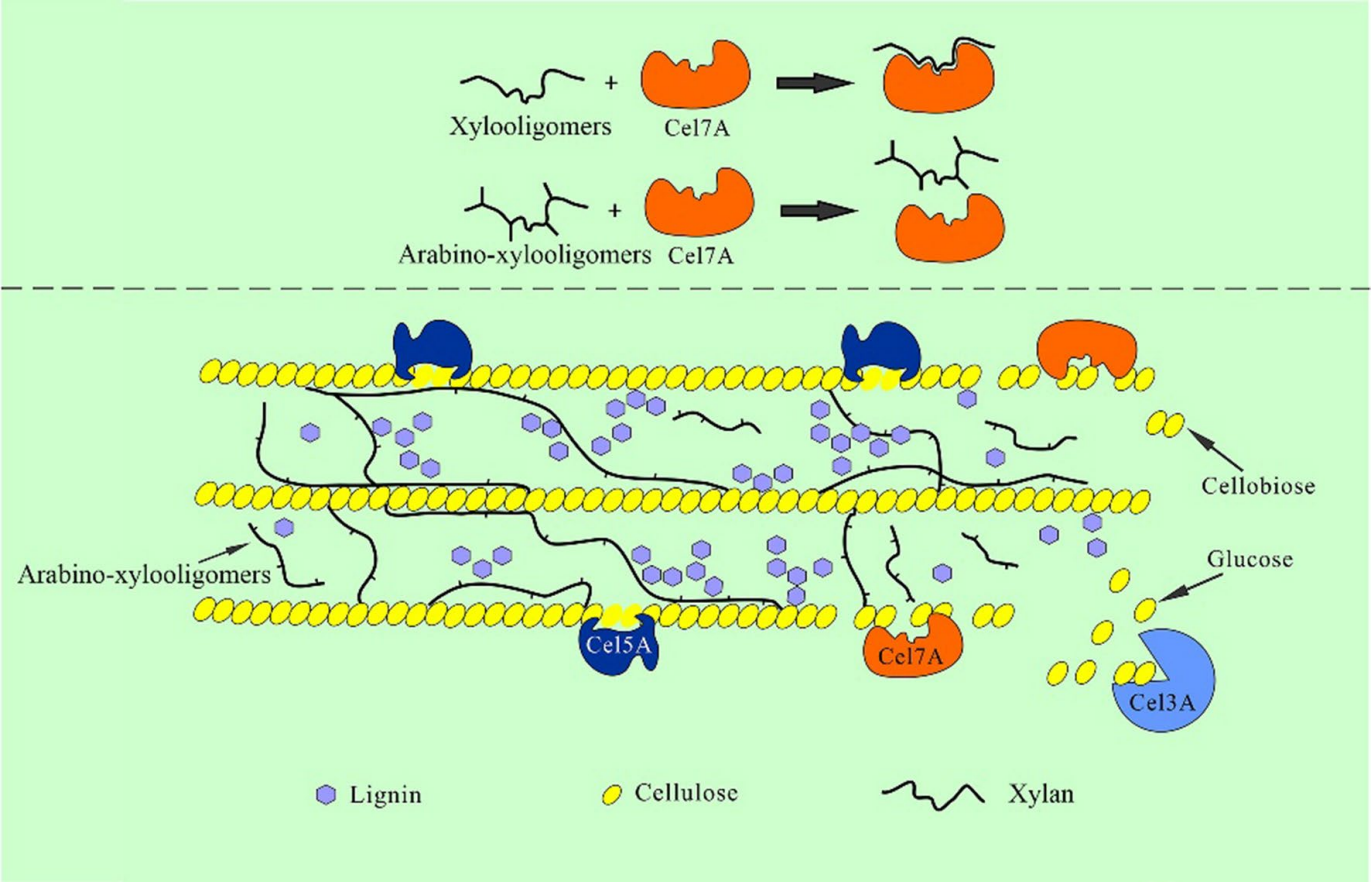
The mechanism behind the increased inhibition. Arabinose substituents on the AXOS backbone block the access of AXOS to the active site of Cel7A

The results presented here clearly show that removal of arabinose substituents from the AXOS backbone can significantly intensify the inhibitory capacity of AXOS on cellulases. We previously hypothesized that the similar β-*O*-1,4-structure of xylooligomers and cellobiose was the reason why xylooligomers could occupy the active site of Cel7A, and thus prevent the cellulose chain from moving into the tunnel of cellobiohydrolase [28]. Based on our hypothesis and the kinetic results presented here, it is conceivable that the arabinose substituents on the AXOS backbone could block the access of AXOS to the active site of Cel7A and lead to reduced inhibition, as shown in the model in Fig. 6. However, it should be noted that the inhibition constant, *K*_*i*_, of xylobiose on Cel7A was found to be 0.98 mM, which was lower than that of xylotriose (3.22 mM) (*P *< 0.01) (Table 3). These results indicated that xylobiose showed a stronger inhibitory effect on Cel7A compared to xylotriose, which is in disagreement with the previous statement that xylooligomers with long chains showed the stronger inhibitory effect compared to those with shorter chains [12, 29]. Here, the difference is likely due to the source of substrate and enzyme. It has been reported that inhibition of cellulases by cellobiose is strongly dependent on the nature of the substrate [36, 37]. In this work, the kinetics of the inhibition of Cel7A was performed with a soluble substrate, which may not apply to the insoluble substrate. In addition, the Cel7A used in this work is from *Thermoascus aurantiacus* and has been heat treated as described in the experimental section. In contrast, the Cel7A used in the work of Baumann et al. is from *Trichoderma reesei* and has not been heat treated. Therefore, the differences in enzyme source and treatment may be another reason for the different inhibition effects caused by xylooligomer substrates of variable length. However, whether the effect of arabinose substituents on AXOS inhibition of cellulases varies with the source of enzyme and substrate needs to be further investigated.

The results revealed the two aspects of ARA in the hydrolysis of arabinoxylan-containing biomass. On the one hand, ARA can cleave arabinose substituents and facilitate effective degradation of arabinoxylan and arabinoxylooligomers, but on the other hand, the addition of ARA may result in more inhibitory, less-substituted xylooligomers as shown in this work. This observation raised the question—how to balance these opposing contributions of ARA and make it play a positive role in biomass hydrolysis.

In Fig. 3, a comparison of AXOS inhibition on the two cellulases tested, that is, CTec2 and CEL, indicated that the addition of ARA showed a stronger effect on enhancing AXOS inhibition on CEL. There are many potential reasons for this phenomenon, but most likely it is associated with the lower xylanase activity, especially endoxylanase and/or β-xylosidase in CEL. Although the addition of ARA resulted in stronger inhibitory, less-substituted AXOS, the less-substituted AXOS was much more easily digested by endoxylanase and β-xylosidase. Higher amounts of endoxylanase and/or β-xylosidase in the CTec2 preparation, thus, resulted in the higher conversion of AXOS into less inhibitory low-molecular-mass AXOS and xylose compared to CEL. This possibility was supported by the observation that addition of ARA increased the hydrolysis of cellulose in AA-CS when increased loadings of XYL were used (Additional file 2: Figure S2). In addition, ARA was used to specifically remove arabinofuranose from singly substituted xylopyranosyl residues, but not from doubly substituted. The arabinofuranose substituents could be totally removed by a combination of ARA used in this work and ARA that can hydrolyze arabinofuranose from doubly substituted xylopyranosyl residues, such as ARA from GH 43 [38, 39]. The non-substituted xylooligomers could be easily hydrolyzed to less inhibitory xylose by endoxylanase and β-xylosidase. Thus, the formation of strongly inhibitory, less-substituted AXOS could be potentially reduced by carefully optimizing enzyme formulations, such as the source of ARA and the ratio of ARA and other xylanases, but the optimal formula needs to be further investigated.

It should also be noted that the removal of other side-chain groups, such as glucuronic acid and acetyl groups, by their respective debranching enzymes, could actually enhance the conversion of cellulose in biomass [11, 40]. We, thus, hypothesize that xylan and xylooligomers with different side-chain groups may show different effects on cellulase hydrolytic action. This detailed mechanism merits further exploration.

## Conclusions

During the enzymatic hydrolysis of the arabinoxylan-containing biomass, we noted that further addition of ARA showed no effect on the enhancement of cellulose digestibility. We found that this phenomenon can at least partly be explained by increased inhibition of cellulases by AXOS after removal of the side-chain arabinosyl groups. Specifically, this less-substituted AXOS may exhibit a stronger tendency to occupy the active site of Cel7A. One strategy to address this issue could be the optimization of the ratio of ARA and other xylanases, such as endoxylanase and β-xylosidase, to achieve the goal of completely converting the arabinoxylan into its end products of xylose and arabinose, thus eliminating the strong inhibition of cellulase by mid-product less-substituted AXOS. In summary, our results offer a new perspective for the role of ARA in biomass hydrolysis and strategies to design cost-effective enzyme cocktails to achieve the economic conversion of arabinoxylan-containing lignocellulosic biomass to fuels and chemicals.

## Experimental section

### Materials

Microcrystalline cellulose (Avicel PH-101) was purchased from Sigma Chemical Co. (St. Louis, MO, USA). Arabinoxylan (P-WAXYRS) was purchased from Megazyme (Bray, Wicklow, Ireland), and its detailed structure is shown in Fig. 2. Pure xylobiose (XX), xylotriose (XXX), 3^2^-α-l-arabinofuranosyl-xylobiose (A^3^X), 2^3^-α-l-arabinofuranosyl-xylotriose (A^2^XX), and 2^3^,3^3^-di-α-l-arabinofuranosyl-xylotriose (A^2+3^XX) were purchased from Megazyme (Bray, Wicklow, Ireland). All other chemicals used in this work were of analytical grade and purchased from Sigma.

Corn stover was collected from a local farm in Yangling, China. The material was milled, sieved through a 60-mesh screen scale, and pretreated with 25% (w/v) aqueous ammonia with a solid to liquid ratio of 1:10 at 50 °C for 24 h. The composition of these pretreated materials was analyzed as described by the National Renewable Energy Laboratory (NREL) [41], and the contents of cellulose, xylan, and lignin in the pretreated material were 52.7%, 21.2%, and 12.6%, respectively. The amount of arabinose groups was 3.3% of the DM.

### Enzymes

Thermostable glycosyl hydrolase (GH) 5 family endoglucanase from *Thermoascus aurantiacus* (Cel5A), thermostable GH 7 family cellobiohydrolase from *T. aurantiacus* (Cel7A), and thermostable GH 3 family β-glucosidase from *Acremonium thermophilum* (Cel3A) [42] were produced in a genetically modified *Trichoderma reesei* strain in which the genes *cbh1*, *cbh2*, *egl1,* and *egl2*, encoding Tr Cel7A, Tr Cel6A, Tr Cel7B and Tr Cel5A, respectively, had been deleted as previously described [43–45]. The enzyme preparation was adjusted to pH 6.0 and treated at 60 °C for 2 h to inactivate the background *T. reesei* enzymes. Because of the high expression level of native *T. reesei* xylanase genes, it is difficult to totally inactivate the xylanase activity. Hence, there are still some xylanolytic enzymes present in the heat-treated enzymes [31]. The thermostable enzyme preparations were kindly provided by Roal Oy (Rajamäki, Finland). The protein was quantified using the Lowry method [46] with bovine serum albumin (Sigma Chemical Co., St. Louis, MO, USA) as the standard.

The commercial enzyme preparation Cellic^®^ CTec2 (Novozymes A/S, Bagsværd, Denmark) was used as a reference commercial cellulases. The activity of the enzyme was 176 FPU/mL (284.8 mg protein/mL) as measured by the IUPAC standard assay. GH 11 endo-xylanase from *Trichoderma viride* (XYL, E-XYTR1, 10.5 mg protein/mL) and GH51 α-l-arabinofuranosidase from *Aspergillus niger* (ARA, E-AFASE, 9.8 mg protein/mL) were purchased from Megazyme (Bray, Wicklow, Ireland).

### AXOS preparation

The AXOS used in this study was prepared by the hydrolysis of arabinoxylan (5%) using endoxylanase (2 mg/g DM) for 1 h. A sample was then withdrawn and boiled for 10 min to stop the enzymatic hydrolysis. Protein and unhydrolyzed arabinoxylan in the hydrolysate were removed using Amicon Ultra centrifuge tubes (Millipore) by centrifugation at 16,280×*g*, 4 °C for 15 min.

### Enzymatic hydrolysis

The hydrolysis of Avicel and corn stover after pretreatment with aqueous ammonia (AA-CS) using cellulase preparations was performed in tubes with a working volume of 3 mL in 50 mM sodium citrate buffer (pH 5.0) containing 0.02% NaN_3_ at 50 °C [47]. The hydrolysis was conducted in a shaking incubator at 200 rpm. The dose of mixed thermostable cellulase preparation (CEL) was 2 mg/g DM Cel5A, 8 mg/g DM Cel7A, and 0.2 mg/g DM Cel3A. The dosage of the reference commercial cellulase was 10 FPU/g DM CTec2. The dosages of XYL and ARA were 2 mg/g DM and 2 mg/g DM, respectively. Samples were withdrawn and boiled for 10 min to stop the enzymatic hydrolysis. After cooling, the samples were centrifuged at 10,000×*g* for 10 min, and the supernatants were analyzed for glucose and xylose.

### Kinetics of the inhibition of Cel7A

Cel7A activity was assayed by the hydrolysis of 1 mg/mL *p*-nitrophenol-D-cellobioside (*p*NPC) in 50 mM sodium citrate buffer at 50 °C for 30 min as described by Deshpande et al. [48]. The reaction was terminated by the addition of 0.5 mL of 2% Na_2_CO_3_. The formation of *p*NP was measured at an absorbance at 410 nm to determine the activity of Cel7A. A kinetic inhibitory analysis of Cel7A was carried out with different concentrations of XX, XXX, A^3^X, A^2^XX, and A^2+3^XX. Lineweaver–Burk plots were applied to monitor the mechanism of inhibition. The kinetic parameters were determined by fitting the data to the appropriate Michaelis–Menten equations and plotting the double reciprocal of the reaction rates and the substrate concentrations as Lineweaver–Burk plots. In addition, Michaelis maximum velocity (*V*_max_), constant values (*K*_*m*_), and inhibitory constant (*K*_*i*_) were evaluated.

### Carbohydrate analysis

The concentrations of the oligomer sugars in the supernatants were analyzed using the procedure published by the NREL [30]. The filtered samples of the supernatants were brought to a 4% acid concentration using concentrated H_2_SO_4_. After incubation at 121 °C for 1 h in an autoclave, the samples were withdrawn and neutralized with CaCO_3_ to a pH of 5–6. The samples were then filtered into HPLC vials to determinate the amount of monosaccharide as described below. The amount of oligomer sugars was calculated by subtracting the monosaccharide concentration of the non-hydrolyzed samples from the total sugar concentration of the acid-hydrolyzed samples.

The amounts of glucose, xylose, and arabinose were determined using an HPLC system (Agilent 1260, Agilent Technologies, USA). The system was equipped with a refractive index detector and an autosampler. An ion-moderated partition chromatography column (Aminex column HPX-87H) with a Cation H micro-guard cartridge was used. The column was maintained at 45 °C with 5 mM H_2_SO_4_ as the eluent at a flow rate of 0.5 mL/min. Before injection, the samples were filtered through 0.22-µm MicroPES filters, and a volume of 10 µL was injected. The peaks were detected by refractive index and were identified and quantified by comparison to the retention times of authentic standards (d-glucose, D-xylose, and D-arabinose).

The glucose, xylose, and arabinose yields in the hydrolysis of the biomass material were calculated as described in NREL LAP-009 [49].

### Statistical analysis

Analysis of variance (ANOVA) was performed at 95% confidence level to compare group means of experimental data in triplicate using Microsoft Excel 07 and GraphPad Prism. Differences with *P* values of 0.05 or less were considered significant.

## Additional files

**Additional file 1: Figure S1.** Effect of arabinose on hydrolytic action of cellulases. Hydrolysis of 2% Avicel by CTec2 (10 FPU/g DM) and CEL (2 mg/g DM Cel5A, 8 mg/g DM Cel7A, and 0.2 mg/g DM Cel3A) with the addition of arabinose (0.2, 1, and 5 mg/mL) at 50 °C for 48 h. The error bars represent the standard error of three independent experiments.

**Additional file 2: Figure S2.** Hydrolysis of 10% corn stover after pretreatment with aqueous ammonia (AA-CS) by CEL (2 mg/g DM Cel5A, 8 mg/g DM Cel7A, and 0.2 mg/g DM Cel3A) (B), and XYL (4 mg/g DM), and/or ARA (2 mg/g DM) at 50 °C for 24 and 72 h. The error bars represent the standard error of three independent experiments.

## Abbreviations

ARA: α-l-arabinofuranosidase
AXOS: arabinoxylooligomers
A^2^XX: 2^3^-α-l-arabinofuranosyl-xylotriose
A^3^X: 3^2^-α-l-arabinofuranosyl-xylobiose
A^2+3^XX: 2^3^,3^3^-di-α-l-arabinofuranosyl-xylotriose
βG: β-glucosidase
CEL: cellulase
Cel7A: cellobiohydrolase
CS-AA: aqueous ammonia-pretreated corn stover
DM: dry matter
EG: endoglucanase
*P*NP: *p*-nitrophenol
*p*NPC: *p*-nitrophenol-d-cellobioside
XX: xylobiose
XXX: xylotriose
XYL: endoxylanase
