# Supplementary material for: Insight into the role of α-arabinofuranosidase in biomass hydrolysis: cellulose digestibility and inhibition by xylooligomers
Source: Biotechnol Biofuels. 2019 Mar 22;12:64. doi: 10.1186/s13068-019-1412-0 (PMC6429694; doi:10.1186/s13068-019-1412-0)
Supplement: Supplementary file 1 — Additional file 1: Figure S1. Effect of arabinose on hydrolytic action of cellulases. Hydrolysis of 2% Avicel by CTec2 (10 FPU/g DM) and CEL (2 mg/g DM Cel5A, 8 mg/g DM Cel7A, and 0.2 mg/g DM Cel3A) with the addition of arabinose (0.2, 1, and 5 mg/mL) at 50 °C for 48 h. The error bars represent the standard error of three independent experiments. [file 13068_2019_1412_MOESM1_ESM.docx]

Insight into the role of α-arabinofuranosidase in biomass hydrolysis: Cellulose digestibility and inhibition by xylooligomers

Donglin Xin, Xiang Chen, Peiyao Wen, Junhua Zhang*

College of Forestry, Northwest A&F University, 3 Taicheng Road, Yangling 712100, Shaanxi, China.

^*^Corresponding author

junhuazhang@nwsuaf.edu.cn

Email addresses

DX: [xindonglin@nwsuaf.edu.cn](mailto:xindonglin@nwsuaf.edu.cn)

XC: [chenxiang@nwsuaf.edu.cn](mailto:chenxiang@nwsuaf.edu.cn)

PW: wenpeiyao@nwafu.edu.cn

JZ: junhuazhang@nwsuaf.edu.cn

Figure S1. **Effect of arabinose on hydrolytic action of cellulases.** Hydrolysis of 2% Avicel by CTec2 (10 FPU/g DM) and CEL (2 mg/g DM Cel5A, 8 mg/g DM Cel7A, and 0.2 mg/g DM Cel3A) with the addition of arabinose (0.2, 1, and 5 mg/mL) at 50ºC for 48 h. The error bars represent the standard error of three independent experiments.
