# Supplementary material for: Insight into the role of α-arabinofuranosidase in biomass hydrolysis: cellulose digestibility and inhibition by xylooligomers
Source: Biotechnol Biofuels. 2019 Mar 22;12:64. doi: 10.1186/s13068-019-1412-0 (PMC6429694; doi:10.1186/s13068-019-1412-0)
Supplement: Supplementary file 2 — Additional file 2: Figure S2. Hydrolysis of 10% corn stover after pretreatment with aqueous ammonia (AA-CS) by CEL (2 mg/g DM Cel5A, 8 mg/g DM Cel7A, and 0.2 mg/g DM Cel3A) (B), and XYL (4 mg/g DM), and/or ARA (2 mg/g DM) at 50 °C for 24 and 72 h. The error bars represent the standard error of three independent experiments. [file 13068_2019_1412_MOESM2_ESM.docx]

Figure S2. Hydrolysis of 10% corn stover after pretreatment with aqueous ammonia (AA-CS) by CEL (2 mg/g DM Cel5A, 8 mg/g DM Cel7A, and 0.2 mg/g DM Cel3A), and XYL (4 mg/g DM), and/or ARA (2 mg/g DM) at 50ºC for 24 (A) and 72 h (B). The error bars represent the standard error of three independent experiments.
